# Undecalcified Bone Preparation for Histology, Histomorphometry and Fluorochrome Analysis

**DOI:** 10.3791/1707

**Published:** 2010-01-08

**Authors:** Tony Goldschlager, Amany Abdelkader, Jeffrey Kerr, Ian Boundy, Graham Jenkin

**Affiliations:** Monash Immunology and Stem Cell Laboratories, Monash University; Anatomy and Developmental Biology, Monash University

## Abstract

Undecalcified bone histology demonstrates the micro-architecture of bone. It shows both the mineralised and cellular components of bone, which provides vital information on bone turnover or bone formation and resorption. This has tremendous importance in a variety of clinical and research applications. It yields beautiful images^1^ and allows for techniques such as fluorochrome assessment and histomorphometry^2^. Fluorochrome analysis is a technique where fluorescent dyes that bind to calcium are injected at a particular time point, which allows for quantification of the amount of mineralisation at that given time. Histomorphometry is a process of bone quantification at the microscopic level.

Performing undecalcified bone histology is technically challenging, particularly with large size specimens. It requires variations in technique from those used in standard paraffin embedded histology. This video illustrates the process of producing good quality sections and demonstrates the technical difficulties and methods with which to overcome them. Specimen preparation, fixation and processing are achieved with a manner similar to other soft tissues, however due to the density and lower permeability of bone considerably longer fixation and processing times are required, often taking several weeks. Embedding is achieved using a supporting medium with similar or equal hardness and density to the bone such as methacrylate- based resins, but unlike paraffin infiltration and embedding, this is an irreversible step. Sectioning can be achieved by grinding which produces a thicker section, which is optimal for studies such as fluorochrome analysis. This is best achieved using a diamond blade on a macrotome. Alternatively, thinner sections can be produced for light microscopy and this is achieved using a sledge microtome with a very sharp blade. The sledge microtome provides the additional strength and stability required for large, hard blocks. Resin embedded sections can be stained with a variety of stains, which are demonstrated.

**Figure:**
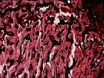


## Protocol

1. The tissue is placed in a sealed container containing 10% phosphate buffered formalin solution^3^. Ensure minimal exposure to light, if fluorochrome analysis is to be performed, as the fluorochromes are light sensitive.
2. Establish which orientation of the tissue will be required.
3. Trim the specimen to size, in the correct orientation, using a band saw. Ensure safety measures are adhered to. The specimen is then placed into an opaque container, the volume of which should be approximately ten times the size of the specimen to achieve adequate fixation. The specimen should remain in the formalin solution a total of between one and two weeks depending on its size. It is suggested using control tissue for testing and optimisation purposes.
4. Check the tissue fits into the embedding mould and place the tissue into a sealed opaque container containing 70% ethanol.
5. Process the tissue in ascending concentrations of ethanol, shielding from light and under constant agitation.
  1. 70 % ethanol 1 week
  2. 80 % ethanol 1 week
  3. 90 % ethanol 1 week
  4. 100 % ethanol 1 week
6. The tissue is then cleared in butanol for 1 week, again shielding from light and under constant agitation.
7. **The density of the resin and bone should be closely matched.** The resin density can be altered (see step 8) and we recommend embedding a test specimen first to ensure correct density. We use Technovit 7100 (Heraeus Kulzer) for light microscopy and fluorochrome analysis. If immunohistochemistry is required an alternative resin must be used such as Technovit 9100 (Heraeus Kulzer).
8. The Technovit 7100 preparation solution may be mixed with small aliquots of the softening solution provided with the kit, if required, but to no more than 5% the volume of preparation solution; in our experience the softening solution is rarely required. Equal parts of absolute ethanol and base liquid Technovit 7100 are mixed and applied to the tissue as a pre-infiltration solution for 24 hours minimising light exposure and with agitation.
9. The infiltration solution is then made up using 1 g hardener I (= 1 bag) which is dissolved in 100 ml base liquid. This replaces the pre-infiltration solution and is allowed to infiltrate for 1 week under constant agitation.
10. Place the tissue into the mould, with the cutting surface face down. Ensure there is a margin (ideally at least 5 millimetres) around the tissue for the resin; this ensures block strength. Mix 15mls of Technovit 7100 preparation solution with 1 ml of hardener II and pour around specimen in the mould and cover it by at least 5 millimietres. Curing will commence within 2 hours but will be complete overnight.
11. Mounting is then performed by fashioning a backing block using Technovit 3040 (Heraeus Kulzer). Mix Technovit 3040 in a volume ratio of 2 parts powder to 1 part liquid to obtain a viscous liquid. Pour Technovit 3040 into the recess at the back of the block to a level of at least 2 mm above the base of the block. After about 10 minutes the mould can be peeled away and the block is ready.
12. The density and therefore the cutting properties of methacrylate resin are susceptible to the ambient humidity in the room. The blocks should therefore be stored in a dessicator.
13. Ground sectioning produces larger sections between 20-50 micrometres in thickness. It is useful for fluorochrome analysis (see section below) as thicker sections produce brighter fluorescence. Secure the diamond blade to macrotome. Add lubricant, such as petroleum spirit to the reservoir on the macrotome. Secure the block in the clamp and orientate to blade. Allow grinding to occur slowly (approximately 20-30 minutes per section) ensuring adequate lubrication. The first section exposes the cutting face and orientates the section- it should be discarded.
14. The next section is then placed on a Superfrost Plus slide. The section has a tendency to curl so we recommend placing a portion of a sandwich bag over it and then holding it flat with another slide clamped in place. It is then placed into an incubator of between 60-80 degrees Celsius for 1 hour. This softens the methacrylate and helps the section adhere to the slide in a flat manner.
15. The ground section may be cleaned and polished as required using fine grit sandpaper in a gentle manner.
16. The sledge microtome cuts thin sections, which provides the best sections for light microsocopy. Use a sledge microtome as it has added rigidity, which is required for cutting these hard blocks. Alternatively a powered rotary microtome can be used.  Use a sharp stainless steel blade. It is preferable to have two blades available as one can be sharpened as the other is used. The blade should make a 45 degree angle with the block. Surface softening can be achieved by applying a wet paper to the block if required. It may take several sections to achieve the desired cutting conditions to obtain a quality section. The section is then floated on a water bath and placed on a slide as in 16 above.
17. Staining reagents are listed in Materials below. Protocols are based on Bancroft^4^ et al. Due to thickness variation it is recommended to optimise stains on test slides. Rack staining may cause tissue to float off the slide so adding the stains directly to a flat slide may be required. An identical protocol or rack staining is necessary for consistent staining outcomes required for histomorphometry.
  1. Haematoxylin and Eosin
    1. Stain with Haematoxyilin 5-10 minutes
    2. Wash well with Scott s tap water
    3. Stain with Eosin 5 minutes
    4. Wash in tap water
    5. Clear in xylene
    6. Mount
    7. Results
      1. Osteoid = pink
      2. Calcified bone = purplish brown
      3. Nuclei = blue
  2. Von Kossa
    1. Place in silver nitrate solution and expose to strong light until mineralised bone turns black (approx 10 mins)
    2. Wash in distilled water three times
    3. Threat with sodium thiosulfate for 5 minutes
    4. Wash in distilled water
    5. Counterstain with Safrinin O
    6. Clear in xylene
    7. Mount
    8. Results
      1. Mineralised bone = black
      2. Osteoid = red/pink
  3. Masson Goldner s Trichrome
    1. Wash with alkaline alcohol (90mls of 80% ethanol and 10mls of 25% ammonia for 20 minutes)
    2. Rinse in water
    3. Rinse in distilled water
    4. Stain with Weigert s Haematoxylin for 10 minutes
    5. Rinse in distilled water
    6. Stain with Ponceau-Fuchsin final solution 5 minutes
    7. Rinse with 1% acetic acid 15 seconds
    8. Stain in phosphomolybdic acid-orange G solution 5 minutes
    9. Rinse with 1% acetic acid 15 seconds
    10. Stain with light green 5 minutes
    11. Rinse with 1% acetic acid 3 changes
    12. Rinse in distilled water
    13. Mount
    14. Results
      1. Mineralised bone = green
      2. Osteoid = red/orange
      3. Nuclei = blue grey
      4. Cartilage = purple
18. Fluorochrome preparations:
19. These are administered by slow intravenous injection, at least two weeks apart.
20. Doses
  1. Calcein Green 10 mg/kg IV
  2. Oxytetracycline   50 mg/kg IV
  3. Alizarin Complexone  30 mg/kg IV
21. Preparation of Calcein Green
  1. One gram of Calcein Green is titrated with 1 M NaOH until dissolved.
  2. pH is adjusted with 1% NaOH until pH = 7.1-7.2.
  3. Filter and keep shielded from the light
22. Preparation of Alizarin Complexone
  1. Three grams of Alizarin complexone is titrated with 1 M NaOH until dissolved.
  2. pH is then adjusted with 1% HCl or NaOH until pH = 7.1-7.2.
  3. Filter and keep shielded from the light
23. Preparation of Oxytetracycline
  1. Oxytetracycline is available as an off the shelf anti-microbial drug. No preparation is required.
